# Prediction of Inflammatory Bowel Disease Course Based on Fecal Scent

**DOI:** 10.3390/s22062316

**Published:** 2022-03-17

**Authors:** Sofie Bosch, Dion S. J. Wintjens, Alfian Wicaksono, Marieke Pierik, James A. Covington, Tim G. J. de Meij, Nanne K. H. de Boer

**Affiliations:** 1 Department of Gastroenterology and Hepatology, AG&M Research Institute, Amsterdam UMC, Vrije Universiteit Amsterdam, 1081 HV Amsterdam, The Netherlands; khn.deboer@amsterdamumc.nl; 2 Department of Gastroenterology and Hepatology, MUMC+, Maastricht University, 6229 HX Maastricht, The Netherlands; d.wintjens@maastrichtuniversity.nl (D.S.J.W.); m.pierik@mumc.nl (M.P.); 3 School of Engineering, University of Warwick, Coventry CV4 7AL, UK; a.wicaksono@warwick.ac.uk (A.W.); j.a.covington@warwick.ac.uk (J.A.C.); 4 Department of Pediatric Gastroenterology, AG&M Research Institute, Amsterdam UMC, Vrije Universiteit Amsterdam, 1081 HV Amsterdam, The Netherlands; t.demeij@amsterdamumc.nl

**Keywords:** inflammatory bowel disease, biomarker, volatile organic compounds

## Abstract

The early prediction of changes in disease state allows timely treatment of patients with inflammatory bowel disease (IBD) to be performed, which improves disease outcome. The aim of this pilot study is to explore the potential of fecal volatile organic compound (VOC) profiles to predict disease course. In this prospective cohort, IBD patients were asked to collect two fecal samples and fill in a questionnaire at set intervals. Biochemically, active disease was defined by FCP ≥ 250 mg/g and remission was defined by FCP < 100 mg/g. Clinically, active disease was defined by a Harvey Bradshaw Index (HBI) ≥ 5 for Crohn’s disease or by a Simple Clinical Colitis Activity Index (SCCAI) ≥ 3 for ulcerative colitis. Clinical remission was defined by an HBI < 4 or SCCAI ≤ 2. Fecal VOC profiles were measured using gas chromatography-ion mobility spectrometry (GC-IMS). The fecal samples collected first were included for VOC analysis to predict disease state at the following collection. A total of 182 subsequently collected samples met the disease-state criteria. The fecal VOC profiles of samples displaying low FCP levels at the first measurements differed between patients preceding exacerbation versus those who remained in remission (AUC 0.75; *p* < 0.01). Samples with FCP levels at the first time point displayed different VOC profiles in patients preceding remission compared with those whose disease remained active (AUC 0.86; *p* < 0.01). Based on disease activity scores, there were no significant differences in any of the comparisons. Alterations in fecal VOC profiles preceding changes in FCP levels may be useful to detect disease-course alterations at an early stage. This could lead to earlier treatment, decreased numbers of complications, surgery and hospital admission.

## 1. Introduction

Ulcerative colitis and Crohn’s disease are chronic gastrointestinal illnesses and combined are referred to as inflammatory bowel disease (IBD). For both phenotypes, prevalence and incidence are high and increasing numbers are being reported globally, with a prevalence of up to 505 UC patients per 100,000 persons in Norway and 322 CD patients per 100,000 persons in Germany [1]. The course of inflammatory bowel disease (IBD) is characterized by periods of relapse and remission. Clinical symptoms often do not correlate with endoscopic and/or radiologic findings and, even during asymptomatic periods, there is often ongoing subclinical inflammation leading to irreversible bowel damage and complications of the disease [2,3].

Insufficient control of (sub)clinical mucosal inflammation leads to irreversible bowel damage and complications [2]. These complications include the formation of circular intestinal scar tissue developing into (obstructive) strictures and internal penetration (e.g., fistula, abscesses), which are often followed by a requirement for surgery (e.g., bowel resection, stricturoplasty, perianal surgery). Deep remission and continuous tight monitoring decrease the rates of complications, need for surgery and hospital admissions [4].

The current gold standard to monitor mucosal inflammation in IBD is ileocolonoscopy. For patients, the burden of frequent colonoscopies is high, and for health care, it is expensive and labor intensive. Therefore, non-invasive markers for the early prediction of changes in disease state are indeed warranted. Some specific gene mutations have been shown to be beneficial for early targeted therapy in only a small group of CD patients [5]. Gut microbial markers and fecal calprotectin (FCP) have been presented as predictive markers for both CD and UC patients; however, up to this point, robust validation is lacking [6,7,8].

A relatively new technique in the field of biomarker research is volatile organic compound (VOC) analysis. These VOCs are the end-products of metabolic pathways in day-to-day life and change in specific situations, such as increased inflammation, cancer degeneration and necrosis. VOCs can be measured in all bodily excrements, including urine, breath, sweat, blood and feces. Fecal VOCs are thought to be the molecular end-products resulting from the interaction between gut microbiota and host metabolism [9].

The analytical methods for VOC measurements can be separated into chemical analytical techniques and pattern-recognition technology. In chemical analytical techniques, such as gas chromatography-mass spectrometry (GC-MS), alterations in the presence and concentrations of specific molecules can be detected [10]. This usually leads to the selection of specific molecules for further biomarker development. With pattern-recognition technologies, diseases are discriminated based on the entire VOC profile of the medium measured [11]. Although individual VOC metabolites cannot be identified, disease-specific patterns can be recognized. These VOC patterns include data on all compounds/molecules detected by the technique. Machine learning methods are then used to create discriminative algorithms, whose diagnostic accuracy usually grows with increasing numbers of the samples measured. The main benefit of pattern recognition is that this technique often allows high diagnostic accuracy to be obtained, as algorithms are based on a combination of various biomarkers. In addition, these types of techniques usually allow fast and low-cost measurements to be performed, which highlights their potential for clinical use.

The potential of fecal VOCs to detect IBD in both adults and children has been the subject of various studies, with promising results [12,13,14,15]. No data on its potential for disease-course prediction have yet been published. The early prediction of changes in disease state adds to timely treatment adjustment, which, consequently, improves disease outcome and prevents drug-related side effects. Therefore, the aim of this pilot study is to explore the potential of fecal VOC patterns to predict IBD course in adults.

## 2. Materials and Methods

### 2.1. Study Participants

This prospective pilot study was performed at Amsterdam University Medical Centers (Amsterdam UMCs) and Maastricht University Medical Center+ (MUMC+). Patients aged 18 years or older with an established diagnosis of IBD based on clinical, endoscopic, histological and/or radiological criteria were eligible for this study. Exclusion criteria were other gastro-intestinal diseases (i.e., celiac disease, adenomas, colorectal cancer) and gastroenteritis prior to inclusion (defined by proven infection with *Salmonella* spp., *Shigella* spp., *Yersinia* spp. *Campylobacter* spp. or *Clostridium* spp. toxins). This study was approved by the Medical Ethical Review Committee of Amsterdam UMCs and MUMC+.

### 2.2. Sample Collection

Samples were collected by Amsterdam University Medical Centers (AMUCs) and Maastricht University Medical Centre (MUMC). For AUMCs, patients with IBD who had collected fecal samples (Stuhlgefäß 10 mL; Frickenhausen, Germany) for a previous study on fecal volatile organic compound (VOC) profiles were asked to take part in this prospective study. Participants collected two samples from the same bowel movement (one for fecal calprotectin levels and one for eNose measurements) and filled in a questionnaire every four months during a period of 1 year between January 2017 and December 2018. Additional sample sets and questionnaires were collected in case of a disease exacerbation. The FCP samples (measured with an ELISA) were sent to the hospital by mail. Samples for VOC analysis were stored in the participants’ own freezer within one hour; this was followed by collection and transportation to the hospital. Transportation was performed by either a researcher (on dry ice), or the participant (using ice packs and/or ice cubes). Samples were stored at −24 °C directly upon arrival at the hospital. Demographic and disease-related data were extracted from electronic files.

For MUMC, between 2009 and 2010, participating IBD patients were asked to collect stool from one bowel movement on the day of a scheduled consult at the outpatient clinic and transport it in fresh condition. This stool sample was stored in the fridge (4 °C) directly upon arrival at the hospital. Two samples were prepared by a researcher on the day of delivery, one for FCP measurements (measured with an FEIA) and one for research purposes. The second sample was stored at −80 °C directly after preparation.

Disease state was classified into biochemical and clinical disease state. Biochemically, active disease was defined by FCP ≥ 250 mg/g and remission by FCP < 100 mg/g. Clinically, active disease was defined by a Harvey Bradshaw Index (HBI) for Crohn’s disease (CD) or by a Simple Clinical Colitis Activity Index (SCCAI) for ulcerative colitis (UC)/IBD unclassified (IBD-U) of ≥5 or ≥3, respectively. Clinical remission was defined by an HBI < 4 or an SCCAI ≤ 2 [16]. Biochemical and clinical disease states were scored for all fecal samples. When two subsequently collected samples met the criteria of active disease and/or remission (either clinical or biochemical), the fecal sample collected first was included for VOC analyses to predict disease state at the second collection.

### 2.3. GC-IMS Instrumentation

In this study, commercial gas chromatography-ion mobility spectrometry equipment (GC-IMS; FlavourSpec^®^; G.A.S., Dortmund, Germany) was used. We have used this method for a number of clinical studies, due to its high sensitivity and rapid analysis time [14]. This instrument is fitted with a CTC auto-sampler to perform controlled injection of a sample into the unit. A GC-IMS instrument combines a GC column with, in this case, a drift-tube ion mobility spectrometer. The sample is first injected into the GC component, which separates the chemical components based on their interaction with the stationary phase coating the column. As individual chemicals elude from the column, they enter the drift-tube IMS, where they are ionized (in our case, with a tritium source); then, they are propelled through the drift tube by a high electric field. In the opposite direction to the ion flow, a buffer gas (nitrogen) is flowed, which collides with the ions. In general, larger ions are struck more than smaller ions, losing more momentum. Thus, the drift time is a function of their mobility in the high electric field and the loss of momentum due to collisions.

### 2.4. Sample Preparation and Volatile Organic Compound Analyses

Samples and data were prepared, analyzed and pre-processed in conformity with our standard operating procedure. In brief, samples were first allowed to thaw for 4 h at room temperature. Then, subsamples of 500 mg were weighted on a calibrated scale, transferred into a glass vial (20 mL headspace vial; Thames Restek, Saunderton, UK) and re-stored in a −24 °C freezer. Subsamples were shipped to School of Engineering, University of Warwick, on dry ice for fecal VOC analyses. The G.A.S. FlavourSpec was fitted with a 30 m SE-54 column with a 0.32 mm inner diameter (ID) (CS Chromatographie Service, Langerwehe, Germany). The instrument was set up with a GC flow rate = 20 mL/min, drift-tube flow rate = 150 mL/min, IMS temperature = 45 °C, GC temperature (fixed) = 45 °C, sample loop = 45 °C and inlet injector = 45 °C. The total analysis time was 8 min. A data-quality process was also undertaken, where all settings, temperatures, flow rates and RIP position were checked. Furthermore, each output file was checked for information content and blanks were used within the run to ensure there was no carry over.

### 2.5. Statistical Analysis

The statistical analysis used is similar to that presented in [14] and was undertaken in ‘R’. In brief, the GC-IMS method creates high-dimensional data but with a significantly lower information content. Every single sample generates around 11 million datapoints. However, all the chemical information is located in the center of the output file; therefore, we are able to crop this central section without losing any useful information. Here, every sample was cropped with the same settings and these setting were chosen based on a visual inspection of the data. Once completed, a threshold was applied which removed background noise, with the value chosen to be two standard deviations above the average background value.

The data were then analyzed using a 10-fold cross validation, whereby the data were split in 10 groups, where 9 groups were used for training and the 10th group was used as a test set. This process was repeated 10 times until every group had been a test set. Within each fold, features that held discriminatory information were identified using a Wilcoxon rank-sum test. In total, 100 features were used, which, through other studies, we found to provide sufficient information content for the majority of applications. Furthermore, we were able to export the location of these features and replot them onto the original output file of the instrument. These features were used to train two models, specifically, Support Vector Machine and Random Forest classification. From the resultant test probabilities, statistical values such as sensitivity and specificity were calculated.

## 3. Results

In total, 280 IBD patients collected 495 fecal samples (292 CD, 197 UC, 6 IBD-U). The number of samples collected per individual varied, as 159 patients provided 1 sample, 65 patients were sampled twice, 34 patients collected 3 samples, 10 patients collected 4 samples, 10 patients collected 5 samples and 2 participants provided 6 and 8 samples. A total of 182 samples were included in this study. The main reason for exclusion was a lack of two subsequent fecal samples meeting the disease-state criteria. Based on clinical activity, 40 patients remained in remission (group-A1), 12 were initially in remission and had an exacerbation at the second collection (group-A2), 30 had ongoing active disease (group-A3) and 14 went from active disease into remission (group-A4). Based on FCP, 41 patients remained in remission (group-B1), 8 had an exacerbation during the second collection (group-B2), 30 had ongoing active disease (group-B3) and 7 went from active disease into remission at the second collection (group-B4). Baseline demographics are given in Table 1.

The inclusion of inflammatory bowel disease patients was performed according to clinical activity scores and biochemical activity scores. Samples were categorized according to disease activity state in the first collected sample and then split into groups based on the disease activity state in the second sample. Only the primary collected sample was used for the exacerbation prediction pilot. In Table 1, the letter A represents classification based on clinical activity indices using the Harvey Bradshaw Index and Simple Clinical Colitis Activity Index. The letter B represents classification based on biochemical activity using fecal calprotectin. The number 1 corresponds to patients who remained in remission, number 2 corresponds to patients initially in remission who went into exacerbation, 3 corresponds to patients who remained in exacerbation and number 4 corresponds to patients initially in exacerbation who went into remission. The majority of the study participants were between 40 and 50 years of age at study inclusion and between 17 and 40 years of age at IBD diagnosis.

### 3.1. GC-IMS Data

**Figure 1:**
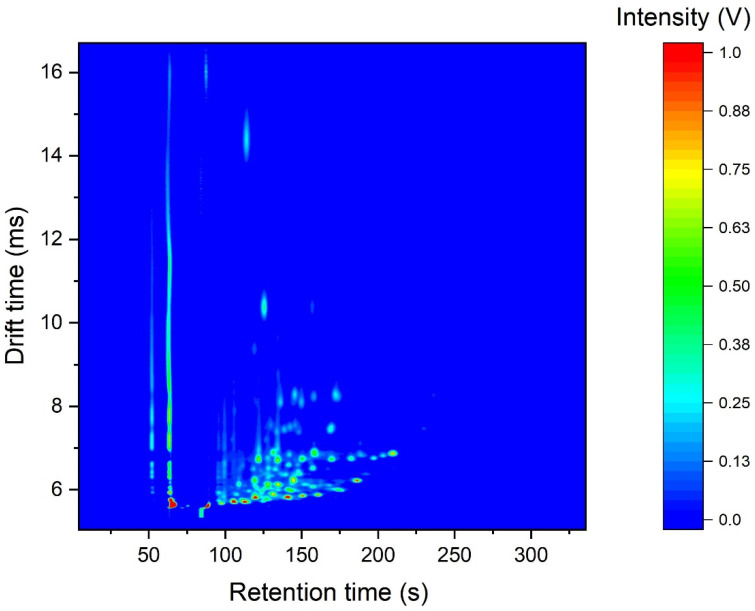
Typical output from a fecal IBD sample as measured with a GC-IMS instrument.

Figure 1 shows a typical GC-IMS output plot for IBD stool samples. In this plot, blue indicates the output when there is no chemical information. The first two vertical lines indicate the output of the instrument to the carrier gas and the white/light blue/red circles indicate individual chemicals separated both by GC and drift-tube IMS. The plot shows that the instrument was able to separate the chemical components in a sample. Furthermore, there was little carry over between samples and the total chemical information per samples is significant, with over a hundred different chemicals having been detected. Furthermore, it shows that the majority of these chemicals were light in nature, as they eluded from the GC at an early point in the analysis.

### 3.2. Prediction of Clinical Disease Course

**Figure 2:**
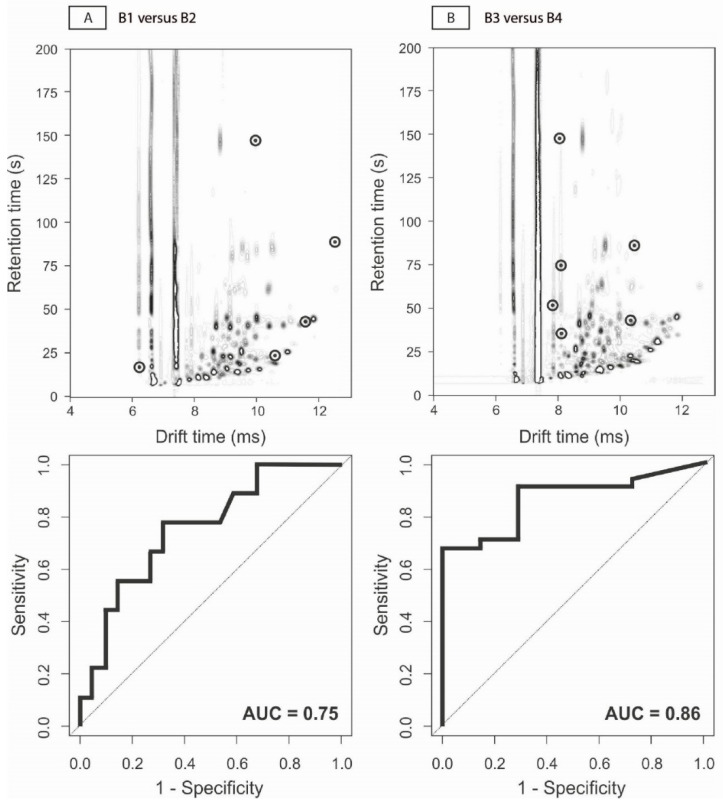
Volatile organic compound profiles and corresponding receiver-operator-characteristic curves. Here depicted, there are example outputs of the gas chromatography-ion mobility spectrometry instrument (GC-IMS; FlavourSpec^®^; G.A.S., Dortmund, Germany). The y-axis represents the retention time in the gas-chromatography column, while the x-axis represents the drift time through the ion mobility spectrometry column. The darkness intensity depicts the level of the measured metabolites. (**A**) depicts an example output of B1 versus B2, i.e., samples of biochemical remission to remission compared with samples of remission to exacerbation. In (**B**), an example VOC output is depicted for B3 versus B4, i.e., samples of biochemical exacerbation to exacerbation compared with samples of exacerbation to remission. In these figures, the bullets mark the locations in the VOC profiles that discriminate cases from controls. Depicted underneath the VOC profiles, there are the receiver-operator characteristic curves and the corresponding areas under the curves for these comparisons.

The results of the VOC analysis by means of GC-IMS are shown in Table 2. Support Vector Machine provided the highest accuracy and its results are depicted in Figure 2. Based on FCP level, the fecal VOC profiles of the remission samples differed significantly between IBD patients prior to exacerbation versus those who remained in remission (B1 vs. B2 AUC 0.75; *p* < 0.01). In addition, samples with high FCP levels at the first time point displayed different VOC profiles in patients preceding remission compared to those whose disease remained active (B3 vs. B4 AUC 0.86; *p* < 0.01). Clinically, based upon disease activity scores, there were no significant differences in any of the comparisons.

## 4. Discussion

In this study, we observed alterations in the fecal VOC profiles of IBD patients preceding a change in the biochemical disease activity parameter fecal calprotectin (FCP). No alterations in fecal VOC profiles were observed preceding a change in clinical disease course as based upon disease activity scores.

There is a high correlation between endoscopic disease activity and FCP when using a cut-off value of 200–250 µg/g [17,18]. Calprotectin itself may be used for the detection of flare-ups and mucosal healing. It has been previously described that IBD patients in remission presenting with increased levels of FCP have an elevated risk of developing a flare-up, whereas mucosal healing can be predicted in patients with decreasing FCP values [19,20]. As alterations in fecal VOC profiles occurred prior to a change in FCP levels in the current study, VOC profiles may be useful markers to predict disease course even at an earlier stage. In the current study, the VOC profiles did not change preceding a change in clinical disease course. This may be explained by the well-known lack of correlation between clinical activity indices and the extent of inflammation [21,22]. Another reason may be the timing of the measurements. Biochemical changes may occur at an early stage of inflammation, whereas symptoms mostly occur when a fulminant inflammation has developed. In this stage, it may be possible that metabolic end-products detected in early stages of inflammation are no longer being produced.

The potential of fecal VOCs to predict biochemical disease course may partly be explained by one of the main sources of fecal VOCs, the gut microbiota [23]. Consisting of over 400 different species, the fecal microbiota has an important role in defending the human body against invading organisms. Although large inter-individual diversity has been reported, the gut microbiome of one individual is remarkably stable [24,25,26]. In IBD patients, the gut microbiome displays a decreased diversity and its composition is more prone to deviations over time [27,28,29,30,31,32]. Additionally, fluctuations in microbiota composition have been observed both during exacerbation and clinical remission of inflammatory bowel disease [27,28,33]. It is possible that the differences in fecal VOC profiles preceding a change in biochemical disease course are a representation of these fluctuations in microbiota composition.

To our knowledge, this was the first study to assess the accuracy of fecal VOCs for the prediction of IBD course. A limitation of the current study was the relatively small sample size that did not allow us to correct for any potential confounders, such as smoking status, BMI, diet and medication use. A larger cohort is required to validate our findings; this may further improve the predictive algorithm. In addition, future studies assessing fecal VOC patterns for prediction of IBD course should focus on endoscopy-controlled cohorts with standardized follow-up moments, ensuring the sole inclusion of patients with active disease and remission based on mucosal appearance and histological findings.

In conclusion, the alterations in fecal VOC profiles preceding changes in FCP levels may be useful to detect disease-course alterations at an early stage. The early detection of changes in disease course would subsequently lead to earlier treatment, decreased numbers of complications, surgery and hospital admission. The results of this pilot study should be confirmed in a larger prospective cohort.

## Figures and Tables

**Table 1:** Demographics.

		A1 (*n* = 40)	A2 (*n* = 12)	A3 (*n* = 30)	A4 (*n* = 14)	B1 (*n* = 41)	B2 (*n* = 9)	B3 (*n* = 30)	B4 (*n* = 7)
Age (y, median (IQR))		42.5 (32–58)	53.5 (42–65.5)	51.5 (43.8–60)	53.5 (43–60.3)	47 (38–57.5)	49 (35–59.5)	39.5 (32–57)	51 (28–66)
Gender (*n* males (%))		22 (55)	6 (50)	7 (23.3)	7 (50)	16 (39)	3 (33.6)	19 (63.6)	2 (28.6)
Smoking status									
Active (*n* (%))		2 (5)	1 (8.3)	4 (13.3)	2 (14.3)	9 (22)	1 (11.1)	1 (3.3)	1 (14.3)
Stopped (*n* (%))		16 (40)	5 (41.7)	13 (43.3)	6 (42.9)	12 (29.3)	4 (44.4)	14 (46.7)	3 (42.9)
Never smoked (*n* (%))		22 (55)	6 (50)	10 (33.3)	5 (35.7)	19 (46.3)	4 (44.4)	15 (50)	2 (28.6)
IBD subtype (*n* CD (%))		24 (60)	9 (75)	21 (70)	12 (85.7)	23 (56.1)	3 (33.3)	16 (53.3)	3 (42.9)
Montreal classification at inclusion									
Age at diagnosis (*n* (%))									
A1	≤16 years	4 (10)	0 (0)	2 (6.7)	1 (7.1)	1 (2.4)	0 (0)	4 (13.3)	0 (0)
A2	17–40 years	22 (55)	7 (58.3)	16 (53.3)	8 (57.1)	27 (65.9)	6 (66.7)	19 (63.6)	4 (57.1)
A3	>40 years	14 (35)	5 (41.7)	12 (40)	5 (35.7)	13 (31.7)	3 (33.3)	7 (23.3)	3 (42.9)
Localization CD (*n* (% of CD))									
L1	Terminal ileum	8 (33.3)	5 (55.6)	7 (33.3)	6 (50)	13 (56.5)	1 (33.3)	2 (6.7)	2 (66.7)
L2	Colon	9 (37.5)	2 (22.2)	3 (14.3)	3 (25)	6 (26.0)	1 (33.3)	5 (31.3)	1 (33.3)
L3	Ileocolic	7 (29.2)	2 (22.2)	9 (42.9)	3 (25)	5 (21.7)	1 (33.3)	9 (56.3)	0 (0)
L4	Involvement Upper GI tract	3 (12.5)	1 (11.1)	5 (23.8)	3 (25)	5 (21.7)	0 (0)	0 (0)	1 (33.3)
Behavior CD (*n* (% of CD))									
B1	NSNP	16 (66.7)	4 (44.4)	11 (52.3)	8 (66.7)	14 (60.9)	1 (33.3)	11 (68.8)	2 (66.7)
B2	Stricturing	4 (16.7)	4 (44.4)	7 (33.3)	3 (25)	7 (30.4)	2 (66.6)	4 (25)	1 (33.3)
B3	Penetrating	4 (16.7)	1 (11.1)	3 (14.3)	1 (8.3)	3 (13.0)	0 (0)	1 (6.3)	0
*p*	Peri-anal	4 (16.7)	3 (33.3)	2 (9.5)	2 (16.7)	5 (21.7)	1 (33.3)	2 (12.5)	0
Extent UC (*n* (% of UC))									
E1	Proctitis	3 (18.8)	1 (33.3)	0 (0)	1 (50)	1 (5.6)	1 (16.7)	3 (21.4)	0
E2	Left-sided	4 (25)	0 (0)	3 (33.3)	0 (0)	9 (50)	0 (0)	4 (28.6)	2 (50)
E3	Pancolitis	9 (56.3)	2 (66.7)	6 (66.7)	1 (50)	8 (44.4)	5 (83.3)	7 (50)	2 (50)

Subject demographics. Abbreviations: IQR, interquartile range; IBD, inflammatory bowel disease; CD, Crohn’s disease; UC, ulcerative colitis; GI, gastro-intestinal; NSNP, non-structuring non-penetrating.

**Table 2:** Differences in fecal volatile organic compound patterns prior to change in disease course.

	AUC (95% CI)	Sensitivity	Specificity	PPV	NPV	*p*-Value
Support Vector Machine classification						
Clinical disease activity						
A1 versus A2	0.62 (0.41–0.82)	0.75	0.58	0.35	0.88	0.89
A3 versus A4	0.53 (0.33–0.72)	0.67	0.50	0.74	0.41	0.62
Biochemical disease activity						
B1 versus B2	0.75 (0.58–0.93)	0.78	0.68	0.35	0.93	0.009
B3 versus B4	0.86 (0.73–0.99)	0.67	1	1	0.41	0.002
Random Forest classification						
Clinical disease activity						
A1 versus A2	0.57 (0.34–0.79)	0.5	0.78	0.4	0.84	0.76
A3 versus A4	0.49 (0.29–0.71)	0.93	0.21	0.72	0.6	0.51
Biochemical disease activity						
B1 versus B2	0.65 (0.41–0.90)	0.67	0.73	0.35	0.91	0.076
B3 versus B4	0.82 (0.68–0.96)	0.63	1	1	0.39	0.004

Based on the training set (70% of the data), the Support Vector Machine and Random Forest classification models were employed using the 100 most discriminatory features and were tested on the test set (30% of the data). The results of the test sets are given in this table. Sensitivities, specificities, *p*-values and AUCs are reported for the respective optimum cut-off points. Abbreviations: A1, from clinical remission to remission; A2, from clinical remission to exacerbation; A3, from clinical exacerbation to exacerbation; A4, from clinical exacerbation to remission; B1, from biochemical remission to remission; B2, from biochemical remission to exacerbation; B3, from biochemical exacerbation to exacerbation; B4, from biochemical exacerbation to remission; AUC, area under the curve; PPV, positive predictive value; NPV, negative predictive value.

## Data Availability

Raw data generated during this study can be accessed via the corresponding authors; data have not been made publicly available online.
